# Cross-Cultural Study of Information Processing Biases in Chronic Fatigue Syndrome: Comparison of Dutch and UK Chronic Fatigue Patients

**DOI:** 10.1007/s12529-017-9682-z

**Published:** 2017-08-23

**Authors:** Alicia M. Hughes, Colette R. Hirsch, Stephanie Nikolaus, Trudie Chalder, Hans Knoop, Rona Moss-Morris

**Affiliations:** 1 Psychology Department, Institute of Psychiatry, Psychology and Neuroscience, King’s College London, London, UK; 2 Expert Centre for Chronic Fatigue, Radboud University Medical Centre, Nijmegen, The Netherlands; 3 Department of Psychological Medicine, Institute of Psychiatry, Psychology and Neuroscience, King’s College London, London, UK; 4 Department of Medical Psychology, Academic Medical Centre (AMC), University of Amsterdam, Amsterdam, The Netherlands

**Keywords:** Chronic fatigue syndrome, Attentional bias, Interpretation bias, Attentional control, Cross-cultural study

## Abstract

**Purpose:**

This study aims to replicate a UK study, with a Dutch sample to explore whether attention and interpretation biases and general attentional control deficits in chronic fatigue syndrome (CFS) are similar across populations and cultures.

**Method:**

Thirty eight Dutch CFS participants were compared to 52 CFS and 51 healthy participants recruited from the UK. Participants completed self-report measures of symptoms, functioning, and mood, as well as three experimental tasks (i) visual-probe task measuring attentional bias to illness (somatic symptoms and disability) versus neutral words, (ii) interpretive bias task measuring positive versus somatic interpretations of ambiguous information, and (iii) the Attention Network Test measuring general attentional control.

**Results:**

Compared to controls, Dutch and UK participants with CFS showed a significant attentional bias for illness-related words and were significantly more likely to interpret ambiguous information in a somatic way. These effects were not moderated by attentional control. There were no significant differences between the Dutch and UK CFS groups on attentional bias, interpretation bias, or attentional control scores.

**Conclusion:**

This study replicated the main findings of the UK study, with a Dutch CFS population, indicating that across these two cultures, people with CFS demonstrate biases in how somatic information is attended to and interpreted. These illness-specific biases appear to be unrelated to general attentional control deficits.

## Introduction

Self-report studies have identified that how people perceive and respond to symptoms, can play a role in maintaining chronic fatigue syndrome (CFS) [1–4]. Experimental studies have explored how people with CFS attend to and interpret illness-related information at earlier, more implicit levels of processing [5–9]. A recent review of this experimental literature concluded that findings were mixed due to a lack of standardized methodology and illness-specific materials, as well as small sample sizes [9].

Subsequent to this review, the authors developed CFS-specific experimental tasks by tailoring materials to tap into concepts central to the illness [10]. A large cross-sectional study (*n* = 103) using these tasks, found people with CFS, showed an attentional bias to information related to fatigue and associated consequences; and tended to form less positive and more somatic interpretations of ambiguous information [11]. Effects were independent of comorbid psychological distress. Contrary to an earlier, smaller study in CFS [6], this larger study found that illness-specific processing biases were not associated with general attentional control deficits.

In order verify these findings, they need to be replicated in another CFS population using the same experimental protocol. Replication is a basic requirement for scientific integrity [12], yet a recent, high profile publication found poor rates of replication success across a range of classic psychological research [13]. Replication in experimental research is particularly pertinent given the range of methodologies employed. Subtle variations in experiments can have implications for the processes which are being tapped into [9]. Furthermore, experimental methods can be prone to error that can arise from errors in millisecond timing and programming [14–16]. Thus, exact replication, using the same experimental protocols, is needed to establish whether findings are reliable and can be extrapolated across populations [14–16].

The aim of this study was to determine whether we can replicate the findings from the UK study [11], with a Dutch CFS cohort to establish whether cognitive processing biases in CFS are a reliable finding across populations and cultures. Experimental data obtained from a newly recruited Dutch CFS cohort were compared to the data from CFS and healthy participants recruited from the UK who took part in our previous study [11]. No study to date has assessed these cognitive processes in a CFS population from outside the UK. Given that self-report studies have identified Dutch and UK CFS participants have similar symptom profiles [17], illness beliefs, and responses to symptoms [18], it was hypothesised that they will also demonstrate similar cognitive biases. Research has also identified that Dutch and UK people with CFS respond similarly to treatments [18]. Given that treatments are often used across cultures, we wanted to assess whether experimentally measured cognitive processes also apply across cultures. Future research can establish whether these cognitive processes are clinically important.

The main hypotheses are the following: (1) the Dutch CFS group will show significant biases and attentional control deficits compared to the healthy control group but equivalent biases and attentional control to the UK group with CFS. (2) Attention and interpretation biases will be independent of levels of anxiety and depression. (3) Attentional control will not moderate attention or interpretation biases.

## Methods

### Participants

Thirty-eight Dutch CFS participants were recruited from a specialist CFS treatment service of the Radboud University Medical Centre. The 38 Dutch CFS participants were compared to the 52 UK CFS participants recruited from CFS services across the UK and 51 UK healthy controls described in the original study [11]. For details of the UK participant recruitment, see the original study [11]. G*Power analysis [19] indicated that to detect an effect of Cohen’s *f* 0.32 (similar to that of the original study), with an alpha of 0.05 and corresponding power of 0.8, a total of 79 participants were needed; thus, 26 per group. Dutch CFS participants were recruited to match the age and gender of UK CFS participants and the resulting sample size was based on the practicalities of how many were able to be recruited. Inclusion criteria were, meeting Centre for Disease Control (CDC) criteria for CFS [20, 21], being over 18 years old, able to read and write Dutch, and not having received psychological treatment for CFS. The Dutch sample included in this study is representative of CFS patients presenting to services across the Netherlands and similar to CFS patients from UK treatment centers. Research comparing patients from the Radboud center to patients from UK CFS services found few differences between these populations in terms of demographics, symptom profile, or treatment outcomes [18].

### Procedures

The Dutch CFS group followed the same protocol as that of the UK CFS and healthy controls [11]. Participants provided demographic information on age, gender, and employment status and completed Dutch versions of questionnaires and experiments in the following order.

### Measurements

#### Questionnaires

*Chalder Fatigue Questionnaire CFQ* [22, 23] was used as a measure of fatigue severity, consisting of 11 items scored 0–3.

*Work and Social Adjustment Scale WSAS* [24] measured everyday functioning, using 5 items (rated 0–8).^1^

*Hospital Anxiety and Depression Scale HADS* [26] measured levels of depression and anxiety, using 14 items.

#### Information Processing Tasks

Materials for the information processing tasks were translated from English to Dutch, back translated to ensure they retained meaning, and piloted with Dutch participants (Appendix).

1. *Visual-probe task* (*VPT*) [27] assessed attentional bias (AB). Participants completed 16 practice trials followed by 96 experimental trials. Each trial starts with a fixation cross in the center of the screen (500 ms), followed by two words (illness-related v. neutral), appearing above and below the fixation. After 500 ms, the words disappear and one is replaced by an arrow. Participants identify the direction of the arrow by pressing “C” for left and “M” for right. AB scores are calculated as the standardized residual (difference) between reaction times (RT) to probes replacing the illness-related stimuli and RT to probes replacing neutral stimuli. Positive values demonstrate an AB to CFS-threatening stimuli.
2. *Recognition task* [28] assessed interpretation bias (IB). Participants read 10 ambiguously phrased scenarios, followed by a short comprehension question. After reading all 10 scenarios, participants are presented with the title of each scenario in turn and asked to rate four new sentences in terms of how similar or dissimilar they are to the original text (1=not at all similar to 4=very similar). The sentences contain a positive interpretation and an illness-related interpretation of the original scenario. Recognition items also include two “foils” or false statements. Foils are included so that not all items are related to the original text, thereby providing greater face validity for the task. For the purpose of this study, we analyzed mean scores on the interpretation items only. An IB index was also calculated as mean similarity ratings of illness-related interpretations minus positive interpretations. Higher scores indicate an increased somatic interpretation.
3. *Attention network task* (*ANT*) [29] assessed general attentional control.^2^ Participants are presented with a string of five congruent (→→→→→) or incongruent (→→←→→) arrows. Participants’ identify the direction of the central arrow by pressing different keys. Attentional control is calculated by subtracting the mean RT on congruent trials from the mean RT on incongruent trials. Higher scores indicate poorer attentional control.

### Analysis

To test whether there was a main effect between groups, separate one-way ANOVAs were conducted with group (Dutch CFS, UK CFS, and healthy controls) as the between subjects factor and AB and attentional control scores as the dependent variables. The means of the IB task were entered into a two-way ANOVA, with group as the between subjects factor and valance (positive and somatic interpretation scores) as the within subjects factor. Post-hoc ANOVAs and *t* tests were used to clarify significant results (hypothesis 1). ANOVAs were rerun with HADS anxiety and depression scores separately as co-variates (hypothesis 2). To examine if attentional control acted as a moderator of AB or IB, an interaction term was created between groups and centered attentional control scores. The interaction term and group were entered as predictor variables in separate linear regressions with AB scores and IB index as the criterion (hypothesis 3).

## Results

### Sample

See Table 1 for the participant characteristics. There were no significant differences between the Dutch CFS, UK CFS, and healthy control cohorts in terms of gender (χ^2^ (2) = 4.56, *p* = .10), employment (χ^2^ (2) = .21, *p* = .90), type of employment (full or part-time), (χ^2^ (2) = .74, *p* = .69), or marital status (χ^2^ (6) = 11.16, *p* = .69, *p* = .08). There was a significant difference between the groups in education attainment (χ^2^ (2) = 24.10, *p* ≤ .001); with more of the Dutch CFS participants in the low education category^3^ than either the UK CFS or UK healthy control groups (both *p* < .05). Sensitivity analysis of subsequent analyses found no difference in effects when controlling for level of education. Healthy participants were significantly younger than either UK CFS participants, *U* = 1025, *p* = .05, or Dutch CFS participants, *t*(63.83) = 2.09, *p* = .04. Age was not correlated with any of the main outcomes so was not controlled for in subsequent analyses. As expected, UK and Dutch CFS groups had significantly higher rates of anxiety, depression, and disability compared to healthy participants (all *p* < .05).

**Table 1:** Demographic variables and scores on self-report measures and information processing tasks for the Dutch and UK CFS patients and UK healthy controls

	Dutch CFS group *n* = 38	UK CFS group *n* = 52	Control group *n* = 51
Age (years M, SD)	40 (13)	39 (12)	34 (10)
Female, *n* (%)	16 (42)	32 (62)	32 (63)
Employed, *n* (%)	24 (65)	36 (69)	35 (69)
Full-time, *n* (%)	13 (34)	20 (38)	22 (43)
Education level: low	27 (71%)	15 (29%)	13 (26%)
Marital status			
Single	13 (34%)	23 (44%)	29 (57%)
Married/living together	24 (63%)	25 (48%)	20 (39%)
Divorced	0	4 (.08%)	1 (.02%)
Widowed	1(.03%)	0	1 (.02%)
CFQ, M (SD)	24.97 (4.05)	26.83 (4.71)	10.7 (3.3)
WSAS, M (SD)	23.6 (7.29)	23.38 (8.81)	0.5 (2.2)
HADS anxiety, M (SD)	6.41 (3.86)	9.96 (4.84)	4.69 (3.43)
HADS depression, M (SD)	9.0 (4.28)	8.44 (4.0)	2.04 (2.38)
Visual-probe task, M (SD)^1^			
Attentional bias score	.28 (1.16)	.22 (.90)	− .20 (.82)
Interpretation bias task, M (SD)			
Somatic interpretations	2.28 (.51)	2.24 (.59)	2.02 (.41)
Positive interpretations	2.69 (.39)	2.74 (.49)	3.05 (.42)
Attentional control task, M (SD)			
RT to congruent trials	652.95 (222.05)	588.86 (136.58)	510.25 (80.34)
RT to incongruent trials	793.20 (236.38)	722.85 (172.48)	615.94 (95.81)
Attentional control score	140.25 (104.02)	133.62 (73.58)	105.70 (50.4)

*CFS* chronic fatigue syndrome, *CFQ* Chalder Fatigue Questionnaire, *WSAS* Work and Social Adjustment Scale, *HADS* Hospital Anxiety and Depression Scale

^1^One Dutch CFS participant had excessive missing data due to errors and outliers (> 3 SD from the group mean) on both the VPT and ANT, this participant was excluded from both these analysis, consistent with other studies. One UK CFS participant was removed as an outlier on the VPT

The Dutch and UK CFS groups had equivalent levels of fatigue (CFQ), *t*(88) = − 1.94, *p* = .06; functioning (WSAS), *t*(88) = .03, *p* = .90; and depression, *t*(88) = .63, *p* = .53. The Dutch CFS group had significantly lower levels of anxiety compared to the UK CFS group, *t*(88) = − 3.71, *p* < .001.^4^

#### VPT: Attentional Bias in UK and Dutch CFS Groups and Healthy Controls

A one-way ANOVA with AB scores showed a significant main effect of group, *F*(2, 136) = 3.46; *p* = .03; η_p_^2^ = .05. Compared to healthy controls, the Dutch CFS group had a significant AB towards illness-related stimuli, *F*(1, 84) = 4.98; *p* = .03; η_p_^2^ = .06. There were no differences in AB scores between Dutch and UK CFS groups, *F*(1, 86) = .07; *p* = .80; η_p_^2^ = .001.

The main effect remained when controlling for anxiety, *F*(3, 136) = 3.25 *p* = .04; η_p_^2^ = .05, but disappeared when controlling for depression, *F*(3, 136) = 1.17; *p* = .31; η_p_^2^ = .02.

#### Recognition Task: Interpretation Bias in UK and Dutch CFS Groups and Healthy Controls

There was a significant group x valence interaction *F*(2, 138) = 16.84, *p < .*001, η_p_^2^ = .20, which remained significant when controlling for anxiety, *F*(3, 136) = 12.60, *p* < .001, η_p_^2^ = .16; and depression *F*(3, 136) = 10.44, *p < .*001, η_p_^2^ = .13.

The Dutch CFS group endorsed positive interpretations significantly less than healthy controls, *t*(87) = − 4.17, *p* < .001, 95% CI (− 54, − .19); and somatic interpretations significantly more than healthy controls, *t*(87) = 2.61, *p* = .01, 95% CI (.06, 45). There was no significant differences between the CFS groups in ratings of somatic, *t*(88) = .36, *p* = .72, 95% CI (− .19, .28) or positive interpretations, *t*(88) = − .71, *p* = .48, 95% CI (− .23, .11).

#### ANT: Attentional Control in UK and Dutch CFS Groups and Healthy Controls

There was a non-significant trend towards a main effect of group on attentional control scores, *F*(2, 138) = 2.72, *p* = .069, η_p_^2^ = .04. Separate ANOVAs indicated the Dutch CFS group had significantly poorer attentional control than healthy participants, *F*(1, 87) = 4.29, *p* = .04, η_p_^2^ = .05; but equivalent attentional control to the UK CFS group, *F*(1, 88) = .126, *p* = .72, η_p_^2^ = .001.

#### Moderating Role of Attentional Control on Attention and Interpretation Biases

A linear regression with AB scores as the criterion and the interaction term and group as predictor variables, found attentional control did not moderate the relationship between group and AB; *b* ≤ .02, SE_b_ = .001, *β* = .31, *t*(136) = .1.58, *p* = .12, 95% CI (− .00,.004). A separate linear regression with IB index as the criterion found attentional control was not a significant moderator of the relationship between group and IB; *b* = − 3.85, SE_b_ = .001, *β* = − .01, *t*(140) = − .05, *p* = .96, 95% CI (− .002, .001).

## Discussion

This is the first CFS study to show replication of illness-specific cognitive biases in a population outside of the UK. These findings indicate cognitive biases in CFS are evident across two different cultural cohorts when using illness-specific materials [12]. In line with our previous study [11], attentional control did not moderate AB or IB, suggesting, attentional control is not a mechanism through which these processes occur. In this study, differences between groups in AB disappeared when controlling for depressed mood using the HADS. This is atypical of depression, where AB is found at longer stimuli presentation durations than used here [30]. Furthermore, the original study found AB was independent of comorbid distress [11], as measured by a clinical interview schedule [31]. These differences may be a reflection of the HADS capturing fluctuating mood whereas the CIS-R assessed clinical psychological comorbidity. A psychometric analysis of the HADS suggests it is best viewed as a measure of distress rather than anxiety and depression per say [32].

By carefully conducting a replication of previous experimental research, this study offers some protection against false positives [13]. Replication in this area is particularly pertinent given that a recent systematic review of experimental studies in CFS found mixed results due to a range of methodologies and unspecific materials [9]. Furthermore, large heterogeneity has been identified in CFS [33]. The successful replication of the original findings indicates that cognitive processing biases seem a robust finding in CFS populations across two cultures.

This study adds to the existing literature by comparing populations with CFS from the UK and Netherlands [18]. Findings indicate cognitive and behavioral factors, including cognitive processing biases, have a role to play in CFS, across cultures. However, the current study is limited by a lack of Dutch healthy control group and differences in baseline characteristics of the CFS populations, namely level of education. However, controlling for level of education had no impact on the main effects observed; thus, it does not appear that differences in education attainment are attributed to the cognitive biases observed. It seems likely that this education difference represents differences in the education system rather than something intrinsically different about these populations. Given the differences in the education systems, the categorization of low and high education levels may have been imprecise. While previous studies show no effects of intelligence on cognitive biases, a more culturally appropriate measure of general intelligence would be useful to include. In addition, a clinical comparison group would be enlightening to further explore whether attention and interpretation biases occur in other fatigued populations or are due to the chronicity of illness. Replication studies such as this pave the way for progress in theory and treatment development. Longitudinal studies should build upon this basic research to explore whether these cognitive processes change over time, following interventions and in comparison to other chronic conditions.

## Appendix

**Table 2:** Rated Dutch stimuli for the visual-probe task

Threat word	Neutral word
vermoeidheid	videobeelden
onuitgerust	bezighouden
uitgeput	vertaald
belasting	afdrukken
Slap	pijl
verzwakt	schuilen
Afgemat	gebraad koolsla
moedeloos	omstreken motorblok
Sloom	place mails
Slopend	lengtes
Strijd	blanke gratis
hoofdpijn	speelgoed
uitputting	platenzaak sleutelgat
Futloos	trommen cijfers
Beperkt	cheques portier
Slaperig	voorruit
Bekaf	poppy depot
lusteloos	honingbij
Zwak	kies
gefrustreerd	statistieken
instorten	negentien
tekortschieten	studievrienden
Zwakte	herder
inspanning	prototype

## Footnotes

1 Both English and Dutch versions of the CFQ [22, 24] and WSAS [22, 23, 25] have been validated for use with CFS populations

2 The attention network task measures three aspects of attention: orientation, altering, and attentional control. For the purpose of this study, we have reported only the trials which correspond to the attentional control score.

3 Since the Dutch and UK education system differ substantially, education levels were categorized as low or high. For the Dutch patients, no education, lower and middle vocational education were considered low and higher education was considered high. For the UK patients, no education or only secondary education were considered low and polytechnic and university education high. This definition was based on [18].

4 Sensitivity analysis found no effect of controlling for HADs anxiety in subsequent analyses.
